# BATF2 in human colorectal cancer

**DOI:** 10.18632/aging.100749

**Published:** 2015-05-15

**Authors:** Zebing Liu, Yu Yang, Xiaoyan Zhou

**Affiliations:** Department of Pathology, Fudan University Shanghai Cancer Center, Shanghai, China

Colorectal cancer (CRC) is one of the leading causes of cancer death worldwide. Better understanding of the molecular mechanisms of CRC pathogenesis is crucial to identify new therapeutic targets and facilitate treatment decisions. The recent work from our laboratory found that BATF2 deficiency promotes progression in CRC via activation of HGF/MET signaling, which potentially provides a promising therapeutic strategy: IFNs in combination with MET inhibitors [1].

*Decreased BATF2 expression predicts poor prognosis in CRC*. It is well known that many oncogenes are hyperactivated and suppressor genes are inactivated when non-tumor tissue progressed to invasive carcinoma. BATF2, a novel tumor suppressor and type I IFN-inducible early response gene, has a broader expression profile that includes hematopoietic and non-hematopoietic tissues [2]. Recent evidence suggests that decreased BATF2 expression is associated with an inferior outcome in hepatocellular carcinoma and oral tongue squamous cell carcinoma [3,4]. Our findings that decreased BATF2 correlates with tumor progression and poor CRC patient prognosis strongly support the notion that BATF2 plays a critical role in controlling tumor initiation and subsequent progression and could be used as a candidate marker for CRC diagnosis as well as prognosis [1].

*BATF2 inactivates HGF/MET signaling in CRC.* HGF/MET signaling is frequently activated in diverse human malignancies; yet the underlying mechanism of HGF/MET activation is not fully elucidated. We first identified BATF2 as an independent factor in cell proliferation inhibition *in vitro* and xenograft formation blockage *in vivo* in CRC. Our findings are consistent with the results of Su *et al*., showing that BATF2 is a potent inhibitory factor that arrests cell growth in prostate cancer, glioma, and melanoma cells [2]. Mechanistically, BATF2 overexpression dramatically impairs activator protein (AP)-1 function via the direct binding with c-Jun (a critical component of AP-1). Coincidently, another report documented that overexpression of AP-1 (c-Jun plus c-Fos) dramatically increased MET promoter activity [5], which was also confirmed in our study. Therefore, a novel pathway comprising BATF2 and c-Jun/AP-1 that inhibits canonical HGF/MET signaling is established. Several studies have shown that activation of HGF/MET signaling promotes the progression of many tumors via PI3K/Akt and/or MAPK/ERK. Our data showed that BATF2 overexpression downregulates MET expression by inhibiting transcription and thereby dephosphorylates Akt in CRC cells. Additionally, the results from immunohistochemistry (IHC) further confirmed our previous findings. MET protein expression detected by IHC was gradually enhanced in tissues from non-tumors that progressed to invasive carcinomas. Furthermore, there was a negative correlation between BATF2 and MET protein expression in CRC [1].

*BATF2 is an IFN-stimulated gene*. Interferons (IFNs), apart from their function as anti-viral infection agents, exert a variety of inhibitory effects on cell growth, apoptosis, and angiogenesis. IFNs exert potent anti-tumor effects and are used clinically either as a mono-therapy or as an adjuvant to chemotherapy for a variety of human cancers. For example, in hairy cell leukaemia and chronic myelogenous leukemia (CML), IFN-α2 reduced marrow infiltration with malignant cells and normalized peripheral haematological parameters. In addition to hairy cell leukaemia and CML, therapeutic efficacy of IFN-α2 in causing at least partial disease regression has been identified in other diverse human malignancies including melanoma, renal cell and bladder cancer, lymphoma, myeloma, and Kaposi's sarcoma [6]. IFNs induce growth inhibition by a variety of pathways that involve many IFN-stimulated genes. BATF2 is one of these genes and can be induced by IFNβ, which indicates that BATF2 may be a key component involved in IFN signaling. Both Su *et al*. and we confirmed that BATF2 expression is induced after IFNβ treatment in melanoma cells and CRC cells. Inhibition of BATF2 by an antisense approach in HeLa cells significantly abrogated IFNβ-induced growth inhibition, thus establishing the role of BATF2 in mediating IFN anti-proliferative action [1,2].

*MET inhibitors and IFNs in CRC therapeutic strategy*. Currently, many targeted approaches to cancer therapy have focused on the reliance of cancer cells on HGF/MET signaling for cell proliferation. Although approaches targeting MET, for instance diverse specific MET inhibitors, monoclonal antibodies and siMET, were developed, and have yielded promising results in preclinical studies, few clinical trials of MET inhibitors have demonstrated the expected therapeutic benefits [7]. This inconsistency raises the possibilities that there are different regulatory mechanisms of HGF/MET signaling in different cancers. Based on our findings that BATF2 can dramatically suppress MET expression and an earlier report that MET inhibitor PF-04217903 only partially inhibited HGF-mediated cell proliferation in CRC cells, we evaluated whether the BATF2 inducer IFN-β in combination with the MET inhibitor PF-04217903 would produce a synergistic effect on cell apoptosis and proliferation in CRC cells. Then, our work validated the efficacy of the combination in inhibiting proliferation in vitro and blocking xenograft formation in vivo in CRC [1].

Taken together, these findings indicate that BATF2 status could be served as a potential biomarker to predict prognosis and IFNs in combination with MET inhibitors may serve as a novel therapeutic strategy for CRC patients.
